# Identifying the Facial Nerve in Parotid Surgeries: How We Do It

**DOI:** 10.22038/ijorl.2020.43760.2446

**Published:** 2021-03

**Authors:** Darwin Kaushal, Abhishek Gugliani, Vidhu Sharma, Amit Goyal, Bikram Choudhury, Kapil Soni

**Affiliations:** 1 *Department of Otorhinolaryngology, All India Institute of Medical Sciences, Jodhpur, Rajasthan.*

**Keywords:** Anatomic Variation, Facial Nerve [anatomy], Parotid, Surgery

## Abstract

**Introduction:**

The facial nerve is an important structure related to parotid gland surgery. Its identification at the time of surgery is critical. Multiple anatomical landmarks have been described to aid in its identification. The objective of this study is to assess whether the tympanomastoid suture is a better surgical landmark than the tragal pointer for identifying the facial nerve while performing parotidectomy.

**Materials and Methods:**

Sixty patients presenting over a period of 3 years from 2016 to 2018 with a parotid swelling without pre-operative facial weakness were included in the study. The average distances between the facial nerve (FN) and the tragal pointer (TP), and the facial nerve (FN) and tympanomastoid suture (TMS) were calculated intra-operatively and compared.

**Results:**

Out of the 60 patients operated, 54 underwent superficial parotidectomy and 6 underwent total conservative parotidectomy. The mean distance between the FN (main trunk) and TP was found to be 18.38 ± 6.85 mm and that between FN and TMS was found to be 2.92 ± 0.6 mm (P<0.0001).

**Conclusion:**

Tympanomastoid suture is a fairly constant and consistent bony landmark to locate the facial nerve during parotid surgeries as compared to the more commonly used cartilaginous tragal pointer. The results of this study can guide surgeons during parotidectomy, to correctly and promptly identify the facial nerve thereby reducing the risk of injury.

## Introduction

The facial nerve is an important structure related to parotid gland surgeries. Injury to the nerve can affect the facial expressions, speaking, closure of eyes and lead to both emotional and psychological trauma to the patient, hence, correct identification and meticulous dissection for anatomic and functional preservation of this nerve during surgery are absolutely necessary.

There are multiple anatomical landmarks in literature to identify the facial nerve. These are: the tragal pointer (TP), the tympanomastoid suture (TMS) line, the posterior belly of digastric muscle, transverse process of axis vertebrae, angle of mandible and the styloid process (1). There is no consensus about the ideal landmark for identifying the facial nerve (2). The tragal pointer is the most widely used landmark during surgery (1-3). Even so, it comprises of soft tissue which is prone for displacement. To avoid damage to the nerve, we need a stable bony landmark that is rigid and reliable, easy to identify and not variable with soft tissue retraction. The TMS is one such landmark. We found that majority of studies that have been carried out to establish a reliable marker of facial nerve are cadaveric studies. In this study, we discuss the method followed by us for identifying the TMS and using it to locate the facial nerve. For purpose of this study, we measured the average distance between FN and TP, and FN and TMS, demonstrating that the latter is a more consistent landmark. The superiority of our study is that it has been carried out on live patients giving more realistic results during surgery. Another strength of our study is that it has been carried out in patients with parotid neoplasms as compared to cadavers with normal parotid anatomy, addressing real time challenges faced by surgeons. 

## Materials and Methods

Institutional Ethical Committee clearance was taken. This is a retrospective observational study carried out over a period of 3 years from 2016 to 2018. All patients with a newly diagnosed swelling of the parotid gland and without a pre-operative facial weakness were included in the study. Those patients who had undergone surgery previously and had a recurrence of the disease or were suffering from pre-existing facial weakness were excluded. A total of 60 patients were thus included. Written and informed consent was taken from the patients to ascertain willingness to participate in the study. The data collected was recorded in MS Excel spreadsheets and analysed using SPSS (version 22; IBM SPSS, Inc, Chicago, Illinois). Surgery was performed under general anaesthesia with the patient supine and head turned to the contralateral side. Panda’s incision was given in all cases for parotidectomy (4). The incision began in the preauricular crease continuing straight downwards, along the tragus. In a modification of the standard incision, it was extended downwards instead of posteriorly, with a gentle anterior curve around 3 cm inferior to the body of the mandible. The parotid tumour was gently dissected from all around. The TMS was identified by palpation and careful dissection was done to locate the FN (main trunk) which lies deep to the suture line.The facial nerve was identified in all cases.

Using Castroviejo callipers (Ortho Max), the shortest distance between the FN and both the TP and TMS respectively, was recorded (^Fig’s 1^,2). After performing parotidectomy and achieving haemostasis, the skin wound was closed in layers.

**Fig 1:**
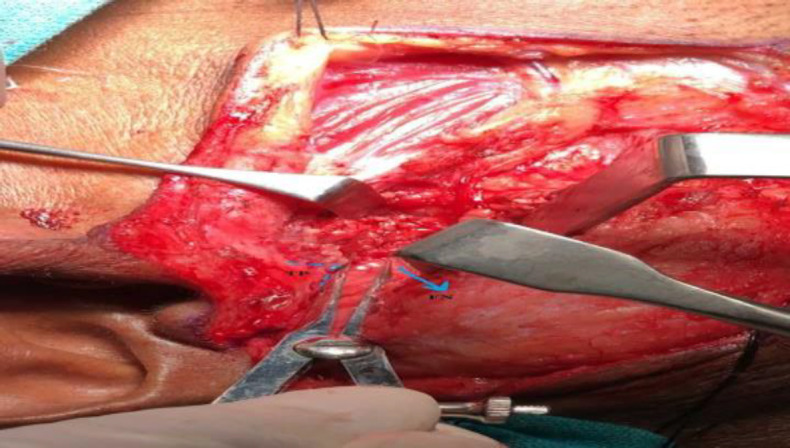
Measuring distance of facial nerve (FN; blue arrow) from tragal pointer (TP; dotted lines)

**Fig 2:**
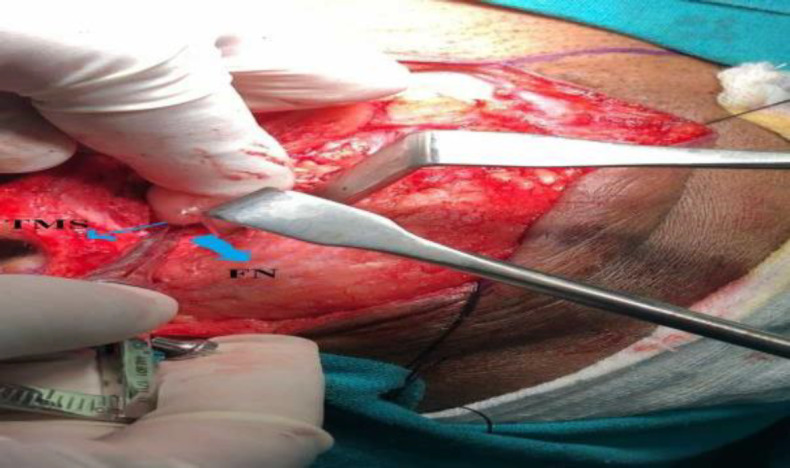
Measuring distance of facial nerve (FN; bold blue arrow) from tympanomastoid suture (TMS; thin blue arrow, identified by palpation)

## Results

Sixty patients were operated. The mean age was 43.75 years. Out of 60 patients, 51 (85%) were males and nine (5%) were females. The mean tumor size removed (in longest dimension) was 40.47 ± 9.39 mm. A superficial parotidectomy was done in 54 patients and a total conservative parotidectomy was done in six patients.

Comparing the distance between the FN and the two landmarks in question, the TMS was found to be a more constant and reliable landmark than TP with a P<0.0001 using the unpaired t-test (Table.1).

**Table 1:** Mean (shortest) distance between FN trunk and the two landmarks

**Mean distance ** **of FN from TMS**	**Mean distance** **of FN from TP**	**P**
2.92 mm ± 0.6 mm	18.38 mm ± 6.85 mm	P< 0.0001

The final histology of the tumor is given in Table 2 and the post-operative complications encountered are shown in Table 3.

**Table 2:** Final Histopathology of the tumor

**Histology**	**Number of Patients (Total = 60)**
Pleomorphic Adenoma	50
Mucoepidermoid Carcinoma	5
Adenoid Cystic Carcinoma	3
Oncocytoma	1
Tuberculosis	1

**Table 3:** Rate of complications

**Complications**	**No. of Patients** **(Total = 60)**
Seroma	45
Temporary facial paresis	3
Marginal mandibular palsy	1
No complication	11

## Discussion

There is controversy and lack of consensus in literature about the intraoperative precision and reliability of various anatomical landmarks for identifying the facial nerve as evident from the large number of studies carried out to find the most precise and consistent one. The landmarks described are the tragal pointer, TMS, posterior belly of digastric muscle, styloid process, mastoid process and peripheral branches of facial nerve (5,6). A review by Ji et el. found the average distance between the FN and TP to be 13.60±11 mm and that between the FN and TMS to be 3.79±2.92 mm (5). 

Rea et el, while studying 26 adult cadavers, found the main trunk of FN to lie at a distance of 10.9±1.7 mm from the TP and 2.5±0.4 mm from the TMS (6). We feel that the results of these studies may not be replicable to live surgical scenarios as these are all cadaveric studies. These studies take into account the cadaveric measurements of soft tissue like the tragal cartilage and the facial nerve which would have undergone contraction thus increasing the measured distances.

The distance between the FN and the TP is variable, being described as 1-3 cm in different studies. It has been the most common landmark used for the identification of the facial nerve (1). However, being mobile, asymmetrical, having a blunt irregular tip and disparity of interpretation of direction of tragal pointer by many surgeons (7), it may not consistently point towards the nerve. 

The TMS has been accepted to be a good landmark for identifying the FN trunk as it is easily palpable, consistent in its position and by leading directly to the stylomastoid foramen, it allows locating the FN close to the foramen.

Nishida and Matsuura reported that when TMS was used as a landmark, the complexity of the surgery was increased as it mandates periosteal elevation around the external auditory canal and inferior dissection in order to approach the anatomical landmark (8). 

Our surgical approach offers a distinct advantage over other methods because we did not lift the periosteum. Instead, the suture line was identified by palpation, avoiding unnecessary dissection. 

The TMS was found to be a more constant and reliable landmark than the TP and also, the standard deviation when using the TMS as a landmark is less than 1 mm thus reducing the chances of iatrogenic trauma to the FN during dissection. 

## Conclusion

A stable landmark decreases the chances of intraoperative injury to the facial nerve. Being a bony landmark, the TMS is more constant in location and less prone to displacement by retraction as compared to the more commonly used tragal cartilage pointer (P<0.0001). 

A slight modification in using the TMS by preserving the periosteum, proposed by the authors, limits unwarranted dissection while maintaining the ease of nerve identification with lesser inter-subject variability as seen with the tragal pointer. The average distances measured in live subjects in the present study can guide surgeons intraoperatively to correctly and promptly identify the facial nerve, thereby reducing the risk of iatrogenic trauma to the nerve and the resulting devastating complications.

## Limitations of the study

- Calculation of distances between FN and TP/TMS is subject to inter-observer variability.
- Other factors may affect the measurements of distances between the FN and the landmarks, such as mandibular atrophy, laterality, gender, racial factors etc.
